# Incidence and risk factors predicting deep venous thrombosis of lower extremity following spinal fractures

**DOI:** 10.1038/s41598-021-82147-x

**Published:** 2021-01-28

**Authors:** Jiangtao Ma, Pei Du, Jin Qin, Yali Zhou, Ningxi Liang, Jinglve Hu, Yingze Zhang, Yanbin Zhu

**Affiliations:** 1 Hebei Orthopedic Clinical Research Center, Third Hospital of Hebei Medical University, Shijiazhuang, Hebei, 050051 People’s Republic of China; 2 Department of Orthopaedic Surgery, Third Hospital of Hebei Medical University, Shijiazhuang, Hebei, 050051 People’s Republic of China; 3 Orthopaedic Institution of Hebei Province, Shijiazhuang, Hebei, 050051 People’s Republic of China; 4 Key Laboratory of Biomechanics of Hebei Province, Shijiazhuang, Hebei, 050051 People’s Republic of China; 5 Chinese Academy of Engineering, Beijing, 100088 People’s Republic of China; 6 Department of Infection Control and Prevention, Third Hospital of Hebei Medical University, Shijiazhuang, Hebei, 050051 People’s Republic of China

**Keywords:** Biomarkers, Epidemiology

## Abstract

The aim of this study was to investigate the presence of preoperative DVT following spinal fracture and the association between the presence of DVT and risk factors. Ultrasonography and blood analyses were performed preoperatively in patients diagnosed with spinal fracture between October 2014 and December 2018. Univariate analyses were performed on the data of demographics, comorbidities, location of injury, spinal cord injury (SCI) grading and laboratory biomarkers. The receiver operating characteristic (ROC) curve analysis was employed to obtain the optimal D-dimer cut-off value for diagnosis. In total, 2432 patients with spinal fractures were included, among whom 108 (4.4%) patients had preoperative DVTs. The average interval between fracture and initial diagnosis of DVT was 4.7 days (median, 2 days), ranging from 0 to 20 days; 78 (72.2%) were diagnosed within 7 days after injury and 67 (62.0%) within 3 days; 19 (17.5%) patients had proximal vein involved and 89 (82.4%) presented in distal veins. Multivariate logistic regression suggested six risk factors independently correlated to DVT, including delay to DUS (in each day) (odds ratio [OR] = 1.11), ASA class III–IV (OR = 2.36), ASIA grade (A/B) (OR = 2.36), ALB < 3.5 g/dL (OR = 2.08), HDL-C < 1.1 mmol/L (OR = 1.68) and d-Dimer > 1.08 µg/ml (OR = 2.49).

## Introduction

Venous thromboembolism, consisting of pulmonary embolism and deep venous thrombosis (DVT), is a major contributor to all-cause inpatient morbidity and mortality, particularly in patients undergoing traumatic injury. A previous survey based on the national fracture database in China reported the incidence of traumatic spinal fractures was 32.80/100,000 person-year^1^. Consistent with the principles of Virchow’s triad, spinal fracture is widely noted as a significant risk factor for DVT due to systematic hypercoagulability, possible injury in vascular endothelium and venous stasis (neurologic deficit or immobilization). Prior studies revealed the prevalence of DVT was from 0.3 to 31% in patients who underwent spinal surgery, which were greatly heterogeneous on study population, follow-up duration and prophylactic strategy^2^. Specifically, traumatic patients with concomitant spinal cord injury had the occurrence rate of DVT as high as 80% without prophylaxis^3^.

Although DVT is extensively studied in patients following major orthopedic surgery (total hip and knee arthroplasty, hip fracture surgery), the explicit guideline of perioperative prophylactic anticoagulation with regard to spinal fracture remains absent. However, in clinical practice, timely and accurate diagnosis can be hardly achieved due to occult characteristics of early DVT, which delays the thrombolysis treatment and presumably results in lethal complications^4–6^. Once DVT is diagnosed, it requires aggressive and prolonged therapeutic anticoagulation, and is often accompanied by the risk of bleeding, recurrence of DVT and significant healthcare cost, which increases the difficulty of perioperative management. Furthermore, a paucity of epidemiologic data on preoperative DVT following spinal fracture still exists, such as the incidence rate, locations and associated risk factors. It is therefore necessary to identify the preoperative DVT and perform timely evaluation by finding some certain predictive indicators.

This retrospective study was designed to investigate the epidemiologic characteristics of preoperative DVT following spinal fracture based on the current diagnostic methodology and treatment algorithms in our institution, which might be conducive to early assessment on risk stratification for DVT. The secondary goal was to determine the risk factors for predicting the presence of DVT from the preexisting comorbidities or initial biomarkers at admission.

## Methods

The retrospective study was performed in accordance with Strengthening the Reporting of Cohort Studies in Surgery (STROCSS) guideline. The ethics committee of Third Hospital of Hebei Medical University approved this research and waived the requirement for informed consent due to the anonymous nature of data. This study included the patients with spinal fractures who underwent spinal surgery in Third Hospital of Hebei Medical University for surgical treatment between October 2014 and December 2018. The follow-up period lasted from injury to either diagnosis of DVT or surgical treatment. All the data were extracted from the electronic medical record system in our institution and retrospective analyses of overall 2432 patients were conducted, consisting of 1597 males and 835 females. Fasting blood analysis, duplex ultrasound (DUS) screening and detailed neurological examination were conducted after admission.

### Inclusion and exclusion

In this study, patients who had spinal fracture and met the following criteria were included as eligible subjects. Inclusion criteria were: patients aged 18 years and older, definitive diagnosis of spinal fracture with complete medical data, presenting within 7 days after injury. Exclusion criteria were: pathological (metastatic) fracture, concomitant fracture at any other location, a history of venous thromboembolism, current oral contraceptive therapy, active malignancy, presence of hyper-coagulopathy or hematological disorders, or recent use of anticoagulants within 3 months for any other condition. Besides, we did not exclude the patients with suspected or diagnostic intraspinal hematoma (ISH), as previous findings suggested no convincing evidence was associated between ISH expansion and early initiation (< 48 h) of chemoprophylaxis^7^.

### Assessment of spinal cord injury (SCI)

Patients with spinal fracture included in this study received detailed neurological examination at admission. The American Spinal Injury Association (ASIA) Impairment Scale is a standardized neurological examination used to assess the severity of sensory and motor levels affected by SCI^8^. This is a 5-level ranking system ranging from A to E, with ASIA Impairment Scale A representing a “complete loss of neural function” and patients who were “neurologically intact” were assigned an ASIA grade of E. In the current study, ASIA grading was grouped into grade A-B and grade C-E based on the presence or absence of motor function below neurologic level.

### DVT detection and prophylaxis

All the patients received DUS screening with Philips Affiniti 50 ultrasonographic machine (Royal Phillips Electronics, Amsterdam, The Netherlands) in bilateral lower limbs. An ultrasonography radiologist who was blind to any hematological results conducted the scanning to detect DVT within the first 48 h after admission, every 3–7 days and when any symptoms suggestive of DVT were exhibited. Therefore, all DVT reported in this study were symptomatic and asymptomatic at the time of diagnosis. Positive DUS results were confirmed with the presence of any signs as followed: direct visualization of intraluminal thrombus, loss of compressibility of the vein, vein dilated with a diameter greater than that of the adjacent artery, blunted or absent flow augmentation and lack of spontaneous flow^9^. Venography was taken if DVT could not be diagnosed or ruled out by the DUS screening. Thrombosis present in the popliteal vein and/or above was defined as proximal DVT, whereas below the popliteal vein as distal DVT. Thrombus occurring both in calf and thigh veins were regarded as a case of proximal DVT. Prophylactic low-molecular-weight heparin (LMWH) was prescribed within the first 24 h after admission unless the spine surgeon determined an emergent need for spinal cord decompression or any other robust contraindication appeared. Intermittent pneumonic compression (IPC) was regularly used if a negative DUS result was reported. Therapeutic anticoagulants or interventional treatment would commence for DVT-positive patients according to our institutional protocol with complete resolution of symptoms.

### Data collection

The comorbidities and demographic data consisted of gender, age, body mass index (BMI), current smoking, alcohol consumption, hypertension, diabetes, chronic heart disease, liver disease, kidney disease, time to DUS after injury, use of prophylactic LMWH and American Society of Anesthesiologists (ASA) classification, American Spinal Cord Injury Association (ASIA) Impairment Scale.

Overnight fasting blood samples were drawn by aseptic venipuncture from an antecubital vein after admission. The biochemical measurements were determined using an automated chemistry analyzer (Beckman Coulter AU 5800, USA). Quantitative measurement of d-dimer was performed using immunochromatographic assay kit. It was analyzed on Wondfo FS-301 Auto-Immunofluorescence Quantitative Analyzer (Xiamen, China). Other hematological values were analyzed on an automated hematological analyzer (Coulter Ac•T 5diff AL Hematology Analyzer, Beckman Coulter, USA) and Automated Blood Coagulation Analyzer (Sysmex CA-1500, Japan). All assays were performed within 60 min after sample collection in the central laboratory of our institution according to the manufacturers’ instructions. Normal ranges for each assay were determined by the laboratory prior to evaluating the study specimens. Hematological biomarkers included total protein (TP) level, albumin (ALB) level, globulin (GLOB), alanine transaminase (ALT), aspartate transaminase (AST), alkaline phosphatase (ALP), gamma-glutamyl transpeptidase (GGT), cholinesterase (CHE), total bile acid (TBA), hypersensitive C-reactive protein (HCRP), lactate dehydrogenase (LDH), hydroxybutyrate dehydrogenase (HBDH), total cholesterol (TC) level, triglyceride (TG) level, high-density lipoprotein cholesterol (HDL-C) level, low-density lipoprotein cholesterol (LDL-C) level, very low-density lipoprotein (VLDL) level, serum sodium concentration (Na^+^), serum chlorine concentration (CL^-^) , white blood cell count (WBC) , neutrophil count (NEU), lymphocyte count (LYM), red blood cell count (RBC), hemoglobin (HGB) level, platelet (PLT), prothrombin time (PT), activated partial thromboplastin time (APTT), fibrinogen (FIB), antithrombin III (AT III), platelet distribution width (PDW), mean platelet volume (MPV), d-dimer level and osmotic pressure (OSM).

### Statistical analysis

Plasma d-dimer is a useful screening parameter for DVT, while its cut-off value greatly varies for the diagnosis of DVT in different populations and settings^10,11^. In this study, receiver operating characteristic (ROC) curve analysis was employed and the area under the curve (AUC) was used to obtain the optimal cut-off value for DVT diagnosis.

SPSS26.0 was used to perform all the statistical analyses (IBM, Armonk, New York, USA). Continuous variables were presented by mean ± standard deviation (SD), and were evaluated by Student *t* test or Mann Whitney *U* test, as appropriate. Categorical data were expressed as number and percentage and were evaluated by Chi-square or Fisher’s exact test, as appropriate. A multivariate logistic regression model was used to explore the independent risk factors predicting the occurrence of DVT, using the stepwise backward elimination method. Variables with *P* < 0.10 were retained in the final model, and the correlation strength is indicated by the odds ratio (OR) and 95% confidence interval (95% CI). The significance level was set as *P* < 0.05. The Hosmer–Lemeshow (H–L) test was used to evaluate the fitting degree of the final model, and a *P* value less than 0.05 represented an acceptable result.

## Results

Totally, 2432 patients with an operatively treated spinal fractures were included, among whom 108 patients had preoperative DVTs, indicating a morbidity of 4.4%. The details on locations of spinal fractures involved in this study were summarized in Table 1, among which the lumbar spine fracture accounted for the largest proportion (60.6%) of the overall cases. The average interval between fracture and initial diagnosis of DVT was 4.7 days (median, 2 days), ranging from 0 to 20 days. In the total 108 patients with DVT, 78 (72.2%) were diagnosed within 7 days after injury and 67 (62.0%) within 3 days. Nineteen (17.5%) patients had proximal vein involved and 89 (82.4%) presented in distal veins. There were 87 cases of DVT presenting in unilateral lower extremity and 21 in bilateral lower limbs. A total of 194 clots were detected by the DUS screening, manifesting an average of 1.80 (range, 1 to 9) thrombi for each patient. To be noted, the number of thrombus considerably decreased along with it occurring proximally. There were 109 clots in peroneal vein, 53 in anterior /posterior tibial vein, 17 in popliteal vein, 6 in femoral vein, 5 in deep femoral vein and 4 in common femoral vein.

**Table 1:** Locations of spine fracture and preoperative incidence of DVT per type.

Location of fracture	No. patients	No. preoperative DVT (%)	
		Proximal	Distal
Cervical fracture	203 (8.3%)	3 (1.4%)	13 (6.4%)
Thoracic fracture	591 (24.3%)	6 (1.0%)	29 (4.9%)
Lumbar fracture	1475 (60.6%)	10 (0.7%)	38 (2.6%)
Cervical-thoracic fracture	5 (0.2%)	0 (0.0%)	1 (20%)
Cervical-lumbar fracture	2 (0.1%)	0 (0.0%)	1 (50%)
Thoracic-lumbar fracture	156 (6.4%)	0 (0.0%)	7 (4.5%)
Total	2432 (100.0%)	19 (0.8%)	89 (3.7%)

**Figure 1:**
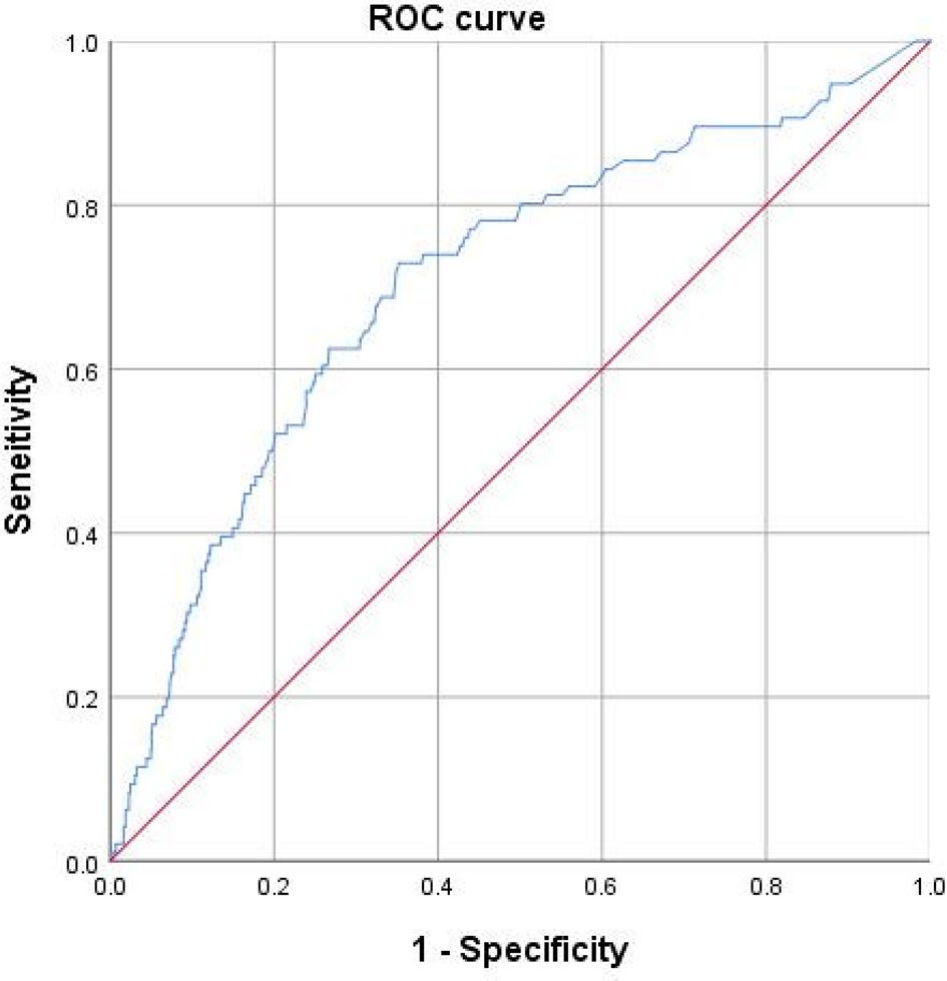
The optimal cut-off for d-dimer for predicting DVT was 1.08 µg/ml, determined by ROC when Youden index was maximum. The area under the curve for plasma d-dimer was 0.707 (95% CI, 0.651 to 0.762; p < 0.001), sensitivity: 72.9, specificity: 64.8.

The optimal cut-off for D-dimer was 1.08ug/ml according to the AUC analysis (Fig. 1). The univariate analysis results suggested the time to DUS between DVT and non-DVT groups were significantly different (10.61 ± 9.0 days vs 4.75 ± 3.9 days, p < 0.001). In addition, current smoking, gender, time from injury to DUS, ASA class, ASIA grade, d-dimer, TP, ALB, ALT, AST, GGT, CHE, LDH, HBDH, HDL-C, Na^+^, WBC, NEU, RBC, HGB, PLT, PDW were tested as significantly different (Table 2). Multivariate logistic regression analysis indicated six variables had significantly independent association with DVT, which were delay to DUS (in each day) (OR = 1.11), ASA class III-IV (OR = 2.36, p < 0.001), ASIA grade A/B (OR = 2.36), ALB < 3.5 g/dL (OR = 2.08), HDL-C < 1.1 mmol/L (OR = 1.68) and d-Dimer > 1.08 µg/ml (OR = 2.49) (Table 3). The Hosmer–Lemeshow test demonstrated good fitness of the final model (X2 = 3.463, p = 0.902; Nagelkerke R^2^ = 0.246).

**Table 2:** Univariate analyses of risk factors associated with preoperative DVT following spinal fracture.

Variables	Number (%) of DVT (n = 108)	Number (%) of non-DVT (n = 2324)	*P*
Gender (male)	86 (79.6)	1511 (65.0)	0.002
Age (years)	49.79 ± 12.7	49.85 ± 14.9	0.958
**BMI (kg/m**^**2**^**)**	25.0 ± 3.8	24.8 ± 3.1	0.443
< 18.5	3 (2.8)	53 (2.3)	0.848
18.5–23.9	38 (35.2)	890 (38.3)	
24.0–27.9	47 (43.5)	1011 (43.5)	
≥ 28.0	20 (18.5)	370 (15.9)	
Diabetes mellitus	17 (15.7)	370 (15.9)	0.960
Hypertension	21 (19.4)	460 (19.8)	0.929
Chronic heart disease	10 (9.3)	201 (8.6)	0.826
Liver disease	4 (3.7)	83 (3.6)	0.942
Kidney disease	3 (2.8)	26 (1.1)	0.120
ASIA (Grade A and B)	28 (25.9)	124 (5.3)	< 0.001
Current smoking	20 (18.5)	270 (11.6)	0.031
Alcohol consumption	5 (4.6)	175 (7.5)	0.260
Time to DUS, day	10.61 ± 9.0	4.75 ± 3.9	< 0.001
LMWH received	102 (94.4)	2135 (91.9)	0.335
**ASA classification**			< 0.001
I and II	61 (56.5)	1780 (80.5)	
III–IV	47 (43.5)	454 (19.5)	
TP (< 60 g/L)	69 (63.9)	805 (34.6)	< 0.001
ALB (< 35 g/L)	67 (62.0)	599 (25.8)	< 0.001
GLOB (< 20 g/L)	27 (25.0)	432 (18.6)	0.096
ALT (> upper limit)	35 (32.4)	321 (13.8)	< 0.001
AST (> upper limit)	36 (33.3)	446 (19.2)	< 0.001
**ALP (U/L)**			0.153
< Lower limit	13 (12.0)	207 (8.9)	
Within normal range	82 (75.9)	1930 (83.0)	
> Upper limit	13 (12.0)	187 (8.0)	
GGT (> 60U/L)	26 (24.1)	286 (12.3)	< 0.001
CHE (< 5 kU/L)	29 (26.9)	247 (10.6)	< 0.001
TBA (> 10 μmol/L)	4 (3.7)	1662 (71.5)	0.432
HCRP (> 8 mg/L)	81 (75.0)	243 (47.7)	0.005
LDH (> 250 U/L)	73 (67.6)	1040 (44.8)	< 0.001
HBDH (> 182 U/L)	64 (59.3)	1100 (47.3)	0.015
TC (> 5.2 mmol/L)	9 (8.3)	371 (16.0)	0.033
TG (> 1.7 mmol/L)	19 (17.6)	353 (15.2)	0.498
HDL-C (< 1.1 mmol/L)	72 (66.7)	994 (42.8)	< 0.001
LDL-C (> 3.37 mmol/L)	13 (12.0)	395 (17.0)	0.178
VLDL (> 0.78 mmol/L)	18 (16.7)	345 (14.8)	0.604
Na+ (< 135 mmol/L)	55 (50.9)	703 (30.2)	< 0.001
CL− (< 99 mmol/L)	20 (18.5)	314 (13.5)	0.139
WBC (> 10 × 10^9^/L)	53 (49.1)	810 (34.9)	0.003
NEU (> 6.3 × 10^9^/L)	74 (68.5)	1267 (54.5)	0.004
LYM (< 1.1 × 10^9^/L)	50 (46.3)	913 (39.3)	0.145
RBC < lower limit	80 (74.1)	1110 (47.8)	< 0.001
HGB < lower limit	82 (75.9)	914 (39.3)	< 0.001
PLT (> 350 × 10^9^/L)	14 (13.0)	111 (4.8)	< 0.001
PT (< 10S)	1 (0.9)	48 (2.1)	0.410
APTT (< 28S)	37 (34.3)	758 (32.6)	0.722
FIB (> 4 g/L)	28 (25.9)	653 (28.1)	0.623
AT III (< 80%)	13 (12.0)	190 (8.2)	0.156
PDW (< 12%)	21 (19.4)	190 (8.2)	< 0.001
MPV (< 7.4fL)	21 (19.4)	324 (13.9)	0.109
d-Dimer (> 1.08 mg/L)	70 (64.8)	746 (32.1)	< 0.001
OSM < 260 mOsm/L	16 (14.8)	217 (9.3)	0.059

*ASA* American Society of Anesthesiologists, *TP* total protein, *ALB* albumin, ALT reference range: female, 7–40 U/L; male, 9–50 U/L, AST reference range: female, 13–35 U/L; male, 15–40 U/L, ALP reference range: female, 35–100 U/L; male, 45–125 U/L, RBC reference range: female, 3.5–5.0 × 10^12^/L; males, 4.0–5.5 × 10^12^/L, HGB reference range: females, 110–150 g/L; males, 120–160 g/L.

**Table 3:** Multivariate analysis of factors associated with DVT following spinal fracture.

Variables	OR	95% CI		*P* value
		Lower limit	Upper limit	
Delay to DUS, day	1.11	1.074	1.138	< 0.001
ASIA (Grade A/B)	3.17	1.841	5.445	< 0.001
ASA class III–IV	2.36	1.527	3.630	< 0.001
ALB (< 3.5 g/dL)	2.08	1.317	3.290	0.002
HDL-C (< 1.1 mmol/L)	1.68	1.068	2.655	0.025
d-Dimer (> 1.08 µg/ml)	2.49	1.596	3.891	< 0.001

## Discussion

The current study, to our best knowledge, is the first investigation that aimed at the preoperative rate of DVT for patients with spinal fractures who received early commencement of thromboprophylaxis and regular use of DUS screening. We found the preoperative incidence of DVT was 4.4% in this certain population, which largely varied from previous findings that basically focused on (ranging 0.3% to 31%)^2^. This great variation could be explained by the heterogeneities in research design, method of surveillance and prophylactic strategy.

Previous literature has well demonstrated physiologic responses that remarkably upregulate plasma procoagulant activity following trauma^12^, which predisposes patients to a hypercoagulable condition. Furthermore, in patients with paraplegia or quadriplegia, venous stasis would deteriorate due to the loss of motor function below the neurologic level. In this study, it is of noted that patients with ASIA (grade A/B) had a 2.36-fold increased risk for the following occurrence of DVT, compared to those graded C–E. The independent association between ASIA (grade A/B) and preoperative DVT was identified in DVT predicting and stratifying among patients with spinal fracture, which was in concordance with the findings by Toker et al.^13^. In conjunction with chemoprophylaxis, timely and regular IPC might optimize the preoperative antithrombotic regimen formulated for such subgroup. Additionally, it is worth conducting a randomized controlled trial to verify the effect of aggressive intervention against DVT and whether there exists a long-term benefit during the neurologic rehabilitation period.

American Society of Anesthesiologists (ASA) classification was originally developed as a potential risk stratification tool that is used to assess patients’ preoperative physical status. In the current study, the ASA class was analyzed as a surrogate of preoperative comorbidities to rank the risk of DVT in patients with spinal fracture. We found that patients with ASA class III-IV had 2.36-fold greater odds of developing DVT over patients with ASA class I-II before surgery. Similarly, the DVT-prediction effect of ASA class was also demonstrated and emphasized in previous studies^14–16^. Although old age was notably related with higher ASA^17^, and older age was a widely-accepted risk factor of developing DVT, we found no significant association between the presence of DVT and older age, which might be due to that older age served as a confounding role in ASA class assignment for this study population. Given many morbidities and ASA class were identifiable after admission, the risk of DVT and other complications related to spinal fracture might be reduced following specific interventions.

Hypoalbuminemia refers to serum albumin level lower than 3.5 g/dL, which is regarded as a marker of malnutrition in trauma patients^18^. Preoperative albumin level has been extensively studied as an important index in the estimation of complications and prognosis for patients undergoing surgery^19–21^. In this study, patients with low albumin levels following spinal fracture had 2.08-fold odds to develop DVT than those within normal albumin level, which was in consistent with the findings reported on other orthopedic population^22^. An increasing body of evidence indicates that hypoalbuminemia triggers hyperfibrinogenemia and platelet aggregability and can be reversed by infusing albumin^23,24^. As a modifiable and independent risk factor, hypoalbuminemia in this population could be improved with therapeutic strategies, although future prospective studies should be conducted to investigate whether the risk of DVT could be reduced by correcting malnutrition after spinal fracture.

In this study, the decreased HDL-C (< 1.1 mmol/L) was associated with the increased risk of DVT (OR = 1.68) after adjustment for confounding caused by other risk factors, which was strongly in line with the previous conclusions reported by Deguchi et al.^25^. Although epidemiological studies and experimental animal models conclude that HDL-C exerts the protection from atherosclerotic cardiovascular diseases^26,27^, its antithrombotic composition and function in venous thromboembolic events are more and more recognized^28^. As for the exact connection between HDL-C level and DVT, several underlying mechanisms have been proposed that HDL-C helps downregulate platelet hyperreactivity, inhibit the coagulation cascade as well as facilitate fibrinolysis^29^. Thus, the low HDL-C level contributes less to coagulation homeostasis in spinal fracture, potentially resulting in a thrombotic tendency. Before well-grounded evidence emerges, low HDL-C level should not be neglected in the stratification of DVT.

Typically, the d-dimer threshold used to determine a positive result was intentionally set low to maximize the sensitivity and reduce false negative rates. However, maximizing sensitivity comes along with lowering specificity^30^. There was a paucity regarding the upper cut-off value of d-dimer in predicting of positive DVT. In the univariate analysis, the pre-set D-dimer cut-off value (0.5 µg/ml) had a notable but statistically nonsignificant association with the subsequent DVT, which reflected that adjusting the threshold according to certain medical condition was warranted^31,32^. According to the results of ROC analysis, the optimal cut-off value of D-dimer was 1.08 µg/ml for the purpose of predicting DVT event, much higher than the pre-set threshold. The final analysis showed that patients with D-dimer level > 1.08 µg/ml had 2.49-fold increased risk for DVT in contrast with its counterparts, independent of presence of other comorbidities. Thus, we assumed that stratifying the risk for DVT based on the adjusted cut-off level seemed to be conducive to increasing the specificity as well as improving the predictive value in spinal fracture.

There was a statistically significant association with the occurrence of DVT and prolonged time between injury and DUS detection. In this study, we found the delay to DUS in each day is independently associated with 11% elevated risk for DVT despite the use of prophylactic anticoagulation. Similarly, Smith et al.^33^ observed a notable correlation between the period of delay and the incidence of thromboembolism event in the population with acute hip or femur fracture. Since all the patients included in this study were screened by DUS within the first 48 h after admission, the difference was mainly due to the referral events. As a tertiary trauma center, our impression was that patients referred from other hospitals (i.e. regional or community hospital) seemed to commence anticoagulant therapy later than the non-referral population. On account of the methodological limitation, it appeared to be unlikely to collect the detailed information on chemoprophylaxis therapy at the referring hospital. Due to the fact that IPC as an adjunctive treatment was initiated after the confirmation of absence of DVT, the late use of IPC may have some correlation with the formation of DVT, particularly in those who are delayed by referrals. Thus, the explicit association between IPC and occurrence of DVT should be further researched. Before the consensus emerges, it was worth considering to perform timely screening for DVT and early initiation of anticoagulation therapy in hospital of all levels for delayed hospitalized patients.

There were several inherent limitations in this study. Firstly, because of the nature of the multivariate analysis, we could hardly include all the variables that might potentially influence the statistical results. Secondly, the relations between the variables and DVT were of association, not of causality. Therefore, the results should be interpreted and integrated with clinical situations. Thirdly, the thromboprophylaxis in referring hospital might make some difference in the following DVTs occurred in our institution, yet it could be hardly captured in our database. Fourthly, to improve the internal validity, we exclude patients with several certain medical conditions (e.g. current oral contraceptive therapy, presence of hyper-coagulopathy or hematological disorders), so our findings may be less applicable to such populations.

## Conclusion

In conclusion, the preoperative incidence of DVT was 4.4% following spinal fracture. Understanding the risk factors, including ASA class III-IV, ASIA grade A/B, ALB < 3.5 g/dL, HDL-C < 1.1 mmol/L, d-Dimer > 1.08 µg/ml and the delay to DUS, helps surgeons to refine the risk stratification profile. In addition, by performing early interdisciplinary management, the DVT rate and associated medical burden can be diminished in patients undergoing spinal surgery, particularly those who are predisposed to it.
